# An adaptive decision-making system supported on user preference predictions for human–robot interactive communication

**DOI:** 10.1007/s11257-022-09321-2

**Published:** 2022-04-09

**Authors:** Marcos Maroto-Gómez, Álvaro Castro-González, José Carlos Castillo, María Malfaz, Miguel Ángel Salichs

**Affiliations:** University Carlos III of Madrid, 15 Butarque Street, Leganés, Madrid Spain

**Keywords:** Autonomous Decision-making, Preference learning, Social Robots, Human–robot interaction, Adaptation, Personalised robotics

## Abstract

Adapting to dynamic environments is essential for artificial agents, especially those aiming to communicate with people interactively. In this context, a social robot that adapts its behaviour to different users and proactively suggests their favourite activities may produce a more successful interaction. In this work, we describe how the autonomous decision-making system embedded in our social robot Mini can produce a personalised interactive communication experience by considering the preferences of the user the robot interacts with. We compared the performance of Top Label as Class and Ranking by Pairwise Comparison, two promising algorithms in the area, to find the one that best predicts the user preferences. Although both algorithms provide robust results in preference prediction, we decided to integrate Ranking by Pairwise Comparison since it provides better estimations. The method proposed in this contribution allows the autonomous decision-making system of the robot to work on different modes, balancing activity exploration with the selection of the favourite entertaining activities. The operation of the preference learning system is shown in three real case studies where the decision-making system works differently depending on the user the robot is facing. Then, we conducted a human–robot interaction experiment to investigate whether the robot users perceive the personalised selection of activities more appropriate than selecting the activities at random. The results show how the study participants found the personalised activity selection more appropriate, improving their likeability towards the robot and how intelligent they perceive the system. query Please check the edit made in the article title.

## Introduction

Social robots with autonomous decision-making capabilities are becoming real in many applications. They are used in tasks related to healthcare (Olaronke et al. 2017; Castillo et al. 2018), education (Bertel and Hannibal 2016), and entertainment (Alonso-Martín et al. 2010). Thus, social robots are intended to coexist with humans at their homes or care centres, requiring robust interaction mechanisms for working during long periods. As Leite et al. (2013) assure, especially ‘in healthcare and therapy, there is a great potential for social robots to assist users over extended periods. Thus, the acceptance and usability of the robot are bound to the accomplishment of their expectations and beliefs (de Graaf et al. 2016). This contribution evaluates groups of socially assistive robots coexisting with their homes and aiding them on different tasks. According to this work, if robots coexist in people’s homes, the adaptation and personalisation of the robot’s behaviour are essential to engage users in the interaction.

In order to achieve adaptation, the robot has to be aware of whom it is interacting with. It must be provided with skills not only to perceive the user presence but to retrieve all the possible information about them (Rossi et al. 2017). Enough information allows roboticists to channel the interaction procedures of the robot towards the needs and requirements of the user. In this line, many works have been developed concerning robot personalisation and adaptation, such as (Tapus et al. 2008; Ahmad et al. 2017; Weber et al. 2018). They agree in presenting a decision-making module embedded in the robot’s interaction architecture and learning mechanisms (mainly supported by reinforcement learning) to accomplish their task. Hence, if the robot behaves during prolonged interactions, a decision-making system and learning mechanisms are essential to attain a personalised, natural, and fluid interaction.

In this study, the main contribution is the development of a preference learning framework that allows a social robot to suggest their preferred activities to each user. The framework integrates into the decision-making system of the social robot Mini (Maroto-Gómez et al. 2018; Salichs et al. 2020) to personalise its autonomous activity selection balancing between selecting the user’s preferred activities and exploring new ones. We believe that personalising the activity selection will improve human–robot communication with the user. Using an online survey, 473 participants provided their defining features and preferences towards the entertaining activities of the robot. Then, a preference learning model estimates the preferences of new users using similar features of the survey participants. The survey contains questions about socio-demographic, habits, interests, and preferences about specific attributes related to our social robot Mini (Salichs et al. 2020) (see Appendix A).

From the variety of preference learning (Fürnkranz J. 2010) techniques, we opted for using label ranking (LR) (Vembu and Gärtner 2010), a supervised method that generates a preference ranking considering a predefined set of items. In the ranking, top-ranked items are preferred, presenting a higher score obtained from a voting process. In this application, the LR algorithm ranks the robot’s activities according to the preferences of each particular user. Thus, we refer to each item in the ranking as a label. Each ranking has a predefined number of items that are the entertaining activities of the robot. Note that unlike most works in preference adaptation, we are not adapting the user’s preferences with the interaction but estimating the possible preferences before interacting, so users do not have to indicate their favourite activities to the robot.

Since initially we lacked information about the best LR method, we reviewed the existing literature about combining LR with random forest classifiers (Breiman 2001). Label ranking forest (LRF) is very promising because it does not need large datasets to produce optimal results like deep learning. Besides, unlike reinforcement learning, LRF does not require long-lasting continuous interaction with the environment, providing accurate and rapid predictions once the model is trained. After reviewing the state of the art in LRF, we compared Top Label as Class (TLAC) (Zhou and Qiu 2018) and Ranking by Pairwise Comparison (RPC) (Fürnkranz and Hüllermeier 2003) to optimise the performance of our framework. Both algorithms are straightforward to implement, require a low computational payload, and yield outstanding results for small datasets. According to previous results (Zhou and Qiu 2018; Fürnkranz and Hüllermeier 2003), Ranking by Pairwise Comparison should perform better in terms of ranking prediction, although it requires intensive training periods. Contrarily, Top Label as Class should compete in performance for those scenarios where the number of items to rank is small, requiring less computational time. In later sections, we present the results that lead us to opt for Ranking by Pairwise Comparison instead of Top Label as Class.

The evaluation of the system started with the selection of the LR algorithm that yielded the best outcomes for our application. Once selected, the second step embraced the design of three case studies showing how the decision-making system of the robot uses the predictions of the preference learning algorithm to balance the activity selection between the user’s preferences and exploring other activities. The balance between activity exploration and exploiting the top-ranked activities is attained by combining proactive selections of the robot with allowing the user to decide which activity to execute. Finally, we conducted a real human–robot interaction experiment to assess whether users prefer a personalised or a stochastic activity selection. Although numerous works present active techniques to learn user preferences using human–machine interactions (Long et al. 2016; Chen et al. 2017; Woodworth et al. 2018; Alkhabbas et al. 2020; Adinolf et al. 2020; Schneider and Kummert 2021; Kubota and Riek 2021), any current works addresses how to estimate the initial preferences of new users that interact with a social robot using data collected from similar users. Besides, any work focuses on proactively suggesting the favourite entertaining activities to improve engagement.

This manuscript reviews, in Sect. 2, the related work that can be found in the literature, focusing on LR preference learning and personalised human–robot interaction. In Sect. 3, we formalise the problem of LRF in terms of Ranking by Pairwise Comparison and Top Label as Class. Section 4 puts forward the core of this manuscript. First, it briefly introduces Mini, the robot we have used in this experiment. Next, we provide a detailed description of its software architecture, standing out the communications of the autonomous decision-making system with the rest of the modules in the architecture. Then, we point out how the algorithms estimate the rankings used for personalising the interaction. The dissertation follows with a description of how the user preferences are managed to balance between activity exploration and selecting the favourite activities of the user. We continue in Sect. 5 with we built the datasets for training the model, the experimental set-up and the evaluation of the system. Section 6 presents the main results of this work. We start with the selection of the best algorithm for our application. Next, we describe three case studies describing the operation modes of the decision-making system. Finally, we show the results of the human–robot interaction study about if the participants rate more positive to interact with a robot with personalised activity selection. Section 7 presents a general discussion about the contribution and the main limitations of the model, and Sect. 8 close this work with a brief conclusion.

## Related work

This section surveys the current state of preference learning for user preference prediction, focusing on LRF algorithms. Additionally, we analyse the most relevant works in adaptive decision-making for social robots, highlighting those used to adapt to each user’s preferences.

### Preference Learning for user preference estimation

*Preference learning* (Fürnkranz and Hüllermeier 2010) is a technique aiming at predicting as output the total or partial rankings of a set of items from training information given as input. Unlike most types of machine learning classifiers, the goal of preference learning is to sort items instead of clustering them (Cohen et al. 1998). Literature usually classifies preference learning in *LR*, *instance ranking* and *object ranking* (Fürnkranz J. 2010). In this work, we work with *LR*, which aims at given an instance space  and a finite set of labels  as input, providing an output space  which defines all total orders of the set of labels for each instance  (Vembu and Gärtner 2010). In this application, we rank a set of labels (activities) presented as total orders for each instance of the input state (ranking of user preferences). For this reason, we use LR to estimate the preferences of each user.

Many methods tackle how to rank preferences as total orders (Fürnkranz J. 2010). These techniques are based on *Utility functions*, *Binary preference relations*, *Model-based* learning, and *Local Rank Aggregation*. Utility functions learn to evaluate individual alternatives assigning a degree of utility to each one (Aiolli and Sperduti 2005). Binary preference relations decompose the learning procedure into binary relations. Pairwise learning (Fürnkranz and Hüllermeier 2003; Fürnkranz et al. 2009) follows two approaches: training a model $M_{ij}$ for each pair of labels expressed in a preference relation of the type $\lambda _{i} >_{x} \lambda _{j}$ for each instance *x* or by training models $M_{i}$ to separate each class $\lambda _{i}$ from class $\lambda _{j}$ being class $\lambda _{i}\ne _{x}\lambda _{j}$. Recently, a new approach focused on learning model-based preference relations assuming a known preference structure is trendy. This approach is less generic because it depends on the particular preference definition. Finally, Local Rank Aggregation (Brinker and Hüllermeier 2006, 2007) generates predictions from nearest-neighbour aggregations from similar estimations. In this dissertation, we compare the performance of Ranking by Pairwise Comparison, which supports learning binary preference relations, and Top Label as Class, which uses Local Rank Aggregation. Both methods integrate random forest to estimate the rankings.

Focusing on LR problems, Vembu and Gärtner (2010) provide a thorough revision of LR algorithms and their applications. Nonetheless, this survey does not contain any reference to LR in combination with random forest (Breiman 2001). Multiple studies address random forest’s benefits in LR problems, especially when the dataset is small. As a starting point in LRF, Aledo et al. (2017) used weak learners based on random forest in their LR tree algorithm. In this regard, De Sá et al. 2017 presented LRF, a method that demonstrated significant results in ranking prediction. Zhou and Qiu (2018) improved de Sá’s LRF by considering top-ranked labels of the rankings in the classifying process, reducing the dimension of the problem. The key of Zhou et Qiu’s algorithm lies in a two-step neighbour rank aggregation performed at leaf nodes. Similarly, Werbin-Ofir et al. (2019) presented a new approximation of LRF classification using voting rules.

LR applications range from information retrieval (Schütze et al. 2008) to search engines and recommender systems (Burke et al. 2011; Bobadilla et al. 2013). Online superstores deeply explored these approaches to increase sales by highlighting preferred products on their websites. These websites employ autonomous decision-making engines which actuate according to feedback obtained from user’s clicking data (Joachims and Radlinski 2007; Zheng et al. 2008). These systems proactively present the favourite products in the first place (usually using a ranking format). In robotics, LR has been used with deep learning to estimate a ranking about the optimal grasping of different objects using images (Han et al. 2019). Similarly, LR has been used to rank affordances of a group of novel objects to assist with manipulation tasks (Chu et al. 2019). Combined with deep learning, label ranking has been broadly used in text classification (Liu et al. 2017; You et al. 2019; Gargiulo et al. 2019; Wang et al. 2021). Numerous works reveal excellent results in LR problems using deep learning models for image classification (Cevikalp et al. 2020; Wu et al. 2020; Wen et al. 2020; Dery 2021; Lei et al. 2021). Finally, LR algorithms with deep learning classification are in information retrieval (Pang et al. 2017), disease diagnosis (Zhou et al. 2021), or in Internet of Things (Alkhabbas et al. 2020).

Although the range of LR applications is extensive, we have not found any work where LR applies to social robotics for estimating user preferences in entertainment domains. Most recent works using LR for preference estimation are deployed in online recommendation systems for superstores websites. Nevertheless, the models presented in these works require extensive training of the preference learning system by the customers by using the superstore’s website for generating an initial set of estimations. Thus, unlike the previous works, we build our datasets from data collected through an online survey, so for training our system any human–robot interaction. In this sense, when the robot meets new users and has to estimate their preferences, it just needs the input vector containing the features of the new user. As described in the following sections, the robot can ask the user to retrieve the information contained in the input vector.

### Robot adaptive behaviour

Autonomous decision-making is an emerging field that is gaining importance in applications such as military (Prelipcean et al. 2010; Goztepe 2015), economics (Parkes and Wellman 2015), or artificial intelligence (Duan et al. 2019). In robots, many works describe systems with autonomous behaviour. Velásquez (1996) developed Cathexis as one of the pioneering works in emotional decision-making for artificial agents. Similarly, Cañamero obtained remarkable results in robot adaptation (Cañamero 1997, 2005). Biologically inspired models mainly work in social robots to improve human–robot interaction emulating human relationships. Considering these works, we pretend to endow the social robot Mini with autonomous activity selection adaptation according to the user’s preferences.

In robot adaptation, Ritschel and André (2017) presented a robot that modulates its personality in real time depending on social cues. In the study, the robot uses reinforcement learning to learn users’ preferences by asking their favourite stories during a storytelling task. Weber et al. (2018) presented a robot that dynamically adapts its humour based on reinforced signals obtained from visual and auditory perceptions of the user, as smiles or laughs. To attain user engagement in social robotics, adaptive mechanisms are essential, as Khamassi et al. (2018) postulate. The authors propose a simulated environment where a robot perceives the user’s engagement, gathering visual information in their study. The robot provides fast adaptation mechanisms modifying its behaviours to maintain the user engaged using reinforcement learning. In scenarios involving long-term child–robot interactions, robot adaptation is essential to maintain engagement (Ahmad et al. 2017). In this work, the NAO robot plays the well-known snakes and ladders game with groups of children. Engagement is achieved by performing game-based, emotional, and memory-based adaptation on real-time interactions. Following a similar line, Martín et al. (2020) presented a novel framework devised for user adaptation and profiling in rehabilitation tasks. Martín et al. describe how a monitoring system controls the patient features and behaviour patterns allowing personalised rehabilitation. Giakoumis et al. (2020) presented a service robot that adapts its behaviour depending on the patient’s mood. Finally, and considering the study presented in (Rosenthal-von der Pütten and Abrams 2020), it is worthy of mentioning that user adaptation may consider the social dynamics of people when operating in a different human environment and the consequences derived from this adaptation. Like in our contribution, Rossi et al. (2017) retrieved information about the robot users to adapt the interaction to them. The authors argue that physical, cognitive, and social information is essential to adapt robot behaviour correctly. Similarly, Martins et al. (2019) review how social robots can adapt to different users but focus on the non-physical component of the interaction. Finally, an extensive review of social robots with adaptive methods in rehabilitation can be found in (Kubota and Riek 2021).

Regarding preference prediction for robots, including social ones, the existing literature is scarce. Similarly to our work, Khalili et al. (2010) proposed an intelligent system for light control, making hierarchical decisions using preferences. Long et al. (2016) proposed a system for predicting and recommending users with their favourite activities in social media. Although the model produces excellent results, it requires the user to interact with the system to estimate future activities. Similarly to the previous contribution, Chen et al. (2017) developed a system for user preference recommendation in online stores. This work presented the drawback of needing repeated interactions with users to start yielding estimations. Woodworth et al. (2018) presented a model that infers the user preferences in assistive tasks. The robot learns these preferences by observing the user behaviour using reinforcement learning. Adinolf et al. (2020) present a robot that learns the preferences of users using their feedbacks in a game agent customisation. The study includes human–robot interaction results showing that designers can apply the users’ preferences in future game versions. In an interesting scenario, a robot learns the favourite actions of the user in the control of a manipulator. Probably, the work with more similarities to this contribution is (Schneider and Kummert 2021). The authors present an exercising scenario where a socially assistive robot learns while interacting with the users suggesting their favourite exercises. In the study, they compare participants’ experiences when encountering an adaptive robot and a general one. The authors conclude that people find the adaptive robot more competent and trustworthy than the general robot. This work tackles preference learning using reinforcement learning since any dataset is available at the beginning of the experiment.

The previous review suggests that most preference learning works in robotics focus on learning by interacting with the system instead of estimating the initial preferences to avoid interacting from scratch. On the one hand, reinforcement learning models require long-lasting interactions to find the real user preferences. Moreover, the finding on the preferences generally occurs from scratch. On the other hand, using deep learning models requires massive datasets to produce accurate results, so we addressed this problem by using random forest methods that do not require such large datasets. Besides, we have not found any work integrating LRF in social robots to produce initial user preferences for entertainment without a previous interaction with the robot. For this reason, this work fills the gap in adapting the human–robot interaction mechanisms of a social robot to recommend their favourite activities, only requiring the users’ features that define the input vector that the model uses for making a preference prediction.

## Label ranking forest for preferences estimation

This section introduces LRF, a method that combines the ranking of items with random forest classification. Then, we briefly describe Ranking by Pairwise Comparison and Top Label as Class, the two LR algorithms compared in this work. Besides, we reason the selection of both methods, enumerating their pros and cons.

### Label ranking forest

Hüllermeier et al. (2008) formulates LR as the prediction, for any instance  in an instance space , of a preference relation of the type  among a finite set of labels , where  means that for each instance , label $\lambda _{i}$ is preferred above label $\lambda _{j}$. Note that the set of labels are ranked according to a total order, defined by a permutation  of $\left\{ 1...m \right\}$, such  whenever $\lambda _{i}\succ _{x}\lambda _{j}$, being $\tau (i)$ the position of $\lambda _{i}$ in the ranking and $\Omega$ the full permutation space. We refer to  as the full set of permutations over *m* labels. Training data instances are presented in the form of  which contains features and labelled rankings in permutation format.

**Fig. 1:**
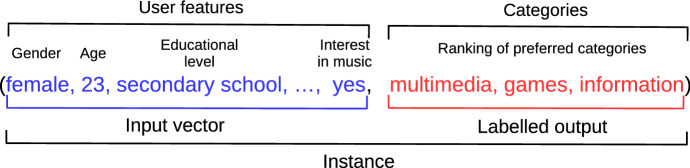
An instance combines the input vector with the labelled output in ranking format. On the one hand, the input vector contains the user’s features regarding the personal, interests, and preferences data about different attributes. On the other hand, the labelled output concerns a predefined set of items (labels) preference ranking. In this case, the example ranks the categories multimedia, games, and information according to the user’s preferences

An instance is each of the inputs of the dataset used for training and validating the model. Each instance has an input vector and a ranking of labels (output). In this work, the input vector is the features of the user. The output is the ranking of activities according to the preferences of the user. The goal of LR is to learn a mapping from , assuming that for every $\tau \in \Omega$ there is a probability  that $\tau$ is the permutation associated with instance . Figure 1 shows the example of an instance in our learning system.

The integration of random forest classifiers (Breiman 2001) in LR algorithms is called LRF (Gharroudi et al. 2014; de Sá et al. 2017; Zhou and Qiu 2018), a method that produces outstanding results even when the dataset is small. Due to the nature of our system, which consists of training various small datasets, we opted for comparing two LRF algorithms to optimise the system’s performance. The following sections introduce Ranking by Pairwise Comparison and Top Label as Class, the two algorithms compared in this study.

### Ranking by pairwise comparison

Ranking by Pairwise Comparison (Fürnkranz and Hüllermeier 2003) splits the classification problem, defined by a set of finite labels , into $m(m-1)/2$ binary classifiers. Each binary classifier  represents a binary preference, for a pair of labels . Thus, each preference relation expressed as $\lambda _{i}>\lambda _{j}$ is converted into a binary comparison where label $\lambda _{i}$ is preferred above $\lambda _{j}$, as Eq. 1 represents.1Consequently, each new model generates, for each instance , a binary label $\in \left\{ 0,1 \right\}$ which represents whether label $\lambda _{i}$ is ranked above $\lambda _{j}$. This preference is stated in the . Once all models make their binary predictions, a voting rank aggregation process produces a ranking where most voted rankings are sorted first. In this work, this ranking sorts the user’s preferences towards a certain list of entertaining activities.

Ranking by Pairwise Comparison simplifies the classification process at the cost of increasing the algorithm’s computational complexity since it trains $m(m-1)/2$ binary classifiers instead of one. Thus, the computational cost of this algorithm increases with the number of labels to rank. Nevertheless, when the number of labels to rank increases, methods like Ranking by Pairwise Comparison based on decoupling the learning problem in several classifiers yield better results in ranking correlation and classification accuracy.

### Top label as class

Top Label as Class is a LRF algorithm developed by Zhou and Qiu (2018). This method replaces the entire rankings found in the labelled output of each instance with its top-ranked label. The top label becomes a new class different from the labels of the ranking. Top Label as Class reduces significantly reduces the label space, simplifying the classification problem. For example, in a domain where the label set is  and the ranking for a particular instance  is $\lambda _{3}>\lambda _{1}>\lambda _{2}$ the full ranking is replaced by its top label, in this case $\lambda _{3}$.

After training the model, a two-step rank aggregation process yields the new ranking predicted by the learning model. The first rank aggregation process uses the arrangements stored in the leaf nodes of each tree in the forest to produce a ranking. Next, the second rank aggregation uses the estimations of each tree in the forest to make a final ranking. Each ranking of a tree equally contributes to the generation of the final ranking. Rank aggregation is tackled using Borda’s method (Brinker and Hüllermeier 2006; Zhou and Qiu 2018; Werbin-Ofir et al. 2019).

Top Label as Class has the advantage of requiring less computational time than, for example, supported vector machines and pairwise comparison, as it just trains one model for the whole problem. However, its main drawback is a significant drop in the performance when the number of labels to rank increases, probably because meaningful information is lost when replacing the full ranking by just their top labels. Top Label as Class and Ranking by Pairwise Comparison have opposite pros and cons. As the following section states, our learning system consists of eight datasets with a variable number of labels to rank, so the selection of both algorithms pretended to optimise the whole system’s performance rather than just one particular dataset. For this reason, the first step in the design of the Preference Learning model was to select the algorithm that yields better results.

## Adaptive activity selection in the social robot mini

**Fig. 2:**
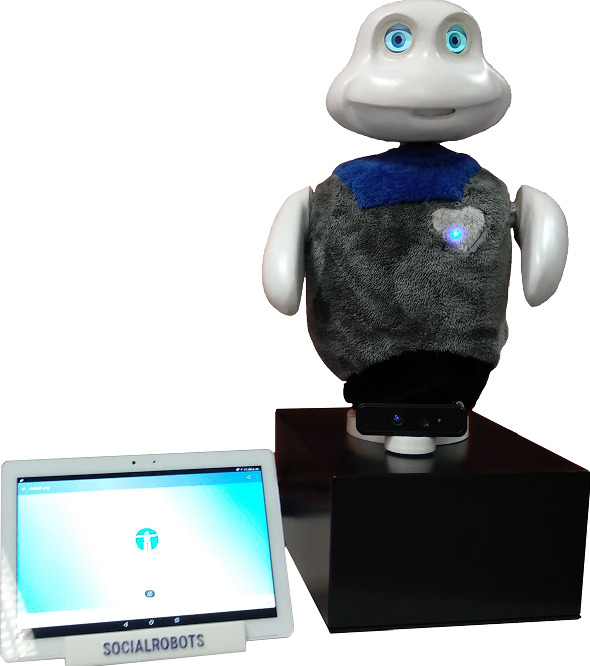
Mini, a social robot designed for entertainment and assisting older adults in cognitive stimulation therapies

In this section, we present the social robot Mini (Salichs et al. 2020), devised for entertainment and assisting older adults in cognitive stimulation therapies. We focus on describing its application to entertainment since it is an important area of social robotics. Figure 2 shows the appearance of the social robot Mini and its touch screen used for entertaining the user.

### Robot architecture

The sensorimotor system of Mini consists of a broad range of sensors and actuators to interact with the environment. Mini contains a 3*D*-stereo camera to detect people and objects, four capacitive touch sensors to detect strokes, and a microphone to understand the user’s speech. Its actuation system contains five degrees of freedom (hip, arms, neck, and head) and four RGB LEDs (cheeks, heart, and mouth). It also has a speaker to communicate and play sounds verbally. Mini communicates with an external touch screen for displaying different games or multimedia content, among other visual and auditory information.

**Fig. 3:**
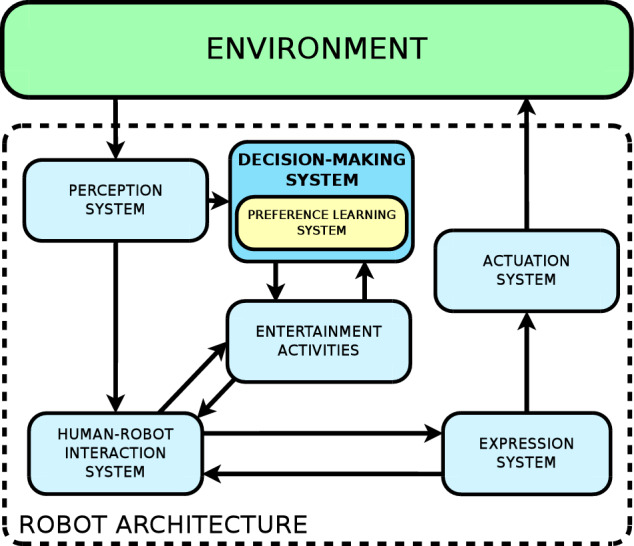
General view of the architecture of the social robot Mini, highlighting its decision-making system module in charged on sequencing the robot’s behaviour

Figure 3 shows the software architecture of the robot. It consists of six modules and different activities which allow it to deploy different functionalities. Communicating with the environment, the perception system perceives its changes, and the actuation system allows the robot to execute physical responses. The task of the perception system is to receive raw data from the robot’s sensors and process them into a message that the other modules in the architecture can understand. On the other hand, the actuation system controls the robot’s actuators and modifies the environment through the execution of specific actions.

The human–robot interaction system (see (Fernández-Rodicio et al. 2020) for further details) controls the interaction with the user. This system receives information from the perception system about environmental changes, process them, and sends an appropriate message to the decision-making system and the robot’s activities. In this manner, the robot produces a suitable interactive communication with the user. Besides, this module handles petitions of other modules like the decision-making system to show or gather information from the user. An example of these petitions is asking the user or display a video on the touch screen. The human–robot interaction system communicates with the expression system, a module that generates appropriate commands to the actuators.

The expression system modulates the expressiveness of the robot in terms of liveliness and emotional gestures. This module executes two main functions. On the one hand, the expression system receives orders (principally from the human–robot interaction system) to control the robot’s actuation. It checks the state of each actuator and controls whether it is possible to execute specific expressions using a priority system. On the other hand, this system receives high-level expressions from the expression scheduler, decomposing them into individual commands to each robot actuator. A particular player dedicates to each actuation unit managing the control of the physical actuator. Finally, the expression system returns essential information to the human–robot interaction system about the execution of each expression.

Finally, the decision-making system is the most critical part of our architecture. It appears highlighted in Fig. 3 since this module contains the preference learning system for adapting the user’s activity selection. The decision-making system decides the activity that Mini executes according to the inputs received from the rest of the modules, using a three-level sequence of decisions (category, subcategory, and activity) balancing between selecting the user’s favourite with exploring new activities. It is worth mentioning that the decision-making system does not always select activities for the user’s entertainment. Mini’s behaviour depends on an internal biologically inspired system (see (Maroto-Gómez et al. 2018)) that represents artificial variables about the physiological and psychological needs of the robot. It is under the activation of the motivational state to play when the preference learning system presented in this manuscript starts working. Once active, it estimates the user’s preferred activities personalising the interaction. When the robot interacts a user, the entertainment motivational state activates more easily, leading to entertainment activities.

The following list introduces and describes the entertaining activities of the social robot Mini. The preference learning system uses these activities to personalise the interaction suggesting the users their preferred ones.

- **Play Bingo! game:** This activity allows the robot to play the well-known Bingo! game with the user.
- **Play a calculus game:** This game consists of the robot asking the user to solve some mathematical operations with different difficulty levels.
- **Play a quiz game:** This game presents the user a set of questions about different topics (e.g. history, science, sports), and the user has to guess the correct answer to win.
- **Weather forecast:** This activity forecasts today’s weather prediction.
- **Informing news:** The robot informs the user about recent news related to some fields as sports, national events, international events, opinion articles, and last-minute information.
- **Play music:** The robot plays songs about a predefined music style (which can be decided by the user or the robot). The available music styles are English pop, English rock, Spanish pop, Spanish rock, Latin music, Classical music, and Flamenco.
- **Display videos:** The robot displays a video using its tablet. The available categories of the video are Sports, Cooking, Famous film trailers, and Comedy.
- **Play audiobooks:** The robot plays an audiobook of the following categories: Famous historical moments, classical books or children’s tales.
- **Play sayings:** The robot tells the user a well-known Spanish saying.
- **Play jokes:** The robot tells the user a funny joke about different topics.
- **Display photos:** The robot displays a group of different photos, which can be about Historical monuments, Incredible landscapes, Animals, or Funny moments.

### Methodology

The adaptive human–robot interaction carried out by the preference learning system is summarised in three steps. First, the entertaining activities of the robot are classified into categories and subcategories. Second, LRF techniques rank the items in each category and subcategory to generate the user preferences. The decision-making system uses these rankings (and associated scores) to carry out a three-step hierarchical decision. The third step embraces the decision itself, leading to the execution of a particular entertaining activity that will usually be among the user’s preferred ones. The decision consists of selecting a category, then a subcategory, and finally an entertaining activity. The entertaining activity must be contained in the subcategory and category selected previously. Instead of directly selecting an activity from the available list, we propose a hierarchical selection that allows the decision-making system to work in different modes.

**Fig. 4:**
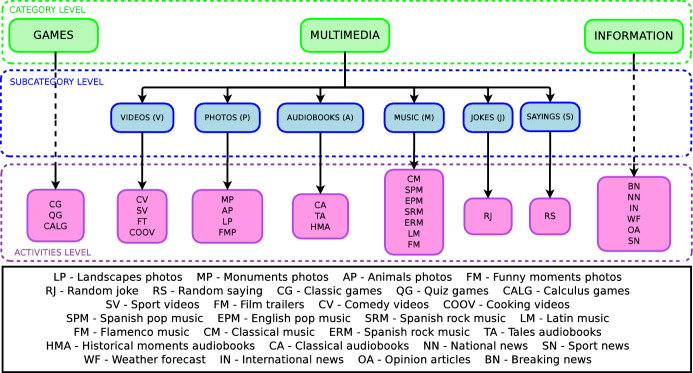
Organisation in three hierarchical levels (categories, subcategories, and entertainment activities) of datasets generated by surveying users. Low-level items are ranked at its higher level producing a ranking of preferences for each user input. In brackets next to each label, its corresponding abbreviation

Figure 4 shows the classification of the entertaining activities of the robot in categories and subcategories. The functionality of each activity denotes in which category and subcategory it is classified. The categories we have defined are: Games, Multimedia, and Information. The Games and Information categories do not have any subcategory directly integrating entertaining activities (types of games and news). The Multimedia category has six subcategories: Videos, Photos, Audiobooks, Music, Sayings, and Jokes. Each of these subcategories contains a group of entertaining activities that the robot can execute. Based on this classification, the preference learning system consists of eight datasets: Entertainment, Games, Multimedia, Information, Videos, Photos, Audiobooks, and Music. The Entertainment dataset ranks the categories Games, Multimedia, and Information according to each user’s preferences. Similarly, the rest of the datasets rank the activities and subcategories using the preferences of each user. For example, the Games dataset ranks the three entertainment activities related to gaming: classical games, quiz games, and calculus games.

Another example is the Videos dataset, which sorts the entertaining activities related to videos displaying: comedy, sports, film trailers, and cooking. Note that like Fig. 4 depicts, each dataset ranks the group of items located in one level below. The translation from the survey data into the eight datasets is described in Sect. 5.1.

Using the ranking defined by each dataset, the decision-making system decides which activity to execute deciding level. The decision-making system works in three modes. These modes combine autonomous decisions made by the robot with decisions from the user. The operating mode depends on the user proactivity level, a parameter gathered by the list of features of the user. The proactivity level can have five numerical values, from 1 to 5. Proactivity values close to 5 represent a user that typically takes the initiative while interacting. Contrarily, proactivity values close to 1 unit indicate a user not taking the initiative while interacting with the robot. The decision-making system works autonomously with users with low proactivity, allows them to select the entertaining activity to execute when encountering very proactive users, and balances both modes for moderate proactive users. We believe that adapting to the proactive level of interacting improves the interaction since the robot aids them if they have difficulties using it.

The autonomous decisions of the robot are carried out using the Boltzmann (Cercignani 1988) distribution, defined in Eq. 2. This distribution balances the selection of preferred entertaining activities with the exploration of new activities assigning a selection probability *p*(*a*) to each alternative. The selection probability depends on the score yielded by the preference learning system (*R*(*a*)), the scores of the other *N* activities, and a parameter in the Boltzmann equation called temperature ($\tau$). The temperature value ranges from 0.1 to 100. A value of 100 units means equal selection probabilities for all labels in the ranking (consequently promoting exploration), whereas values close to 0.1 prioritise the selection of preferred activities by assigning them a higher selection probability. The decision-making system includes mechanisms to avoid repeating the selection of the same activity. By default, the temperature value is 0.1, favouring the selection of the preferred activities of the user. Nevertheless, once a while, the temperature is set to 100 to select the entertaining activity randomly.

2 $$\begin{aligned} p(a) = \frac{e^{\frac{R(a)}{\tau }}}{\sum _{b=1}^{N} e^{\frac{R(b)}{\tau }}} \end{aligned}$$

**Fig. 5:**
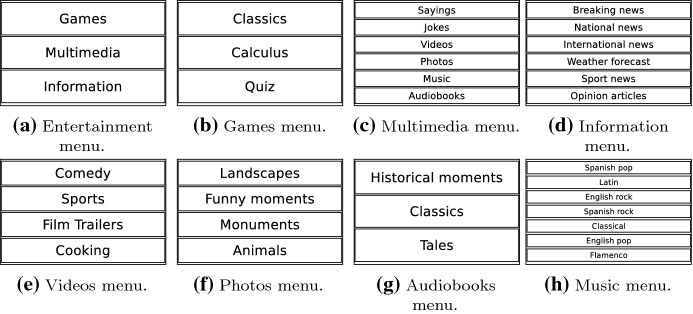
Set of tablet menus shown by the robot giving the users the possibility to select the option they prefer

The decision-making system lets proactive users decide which entertaining activity to execute using tablet menus. Figure 5 shows the tablet menus that can be displayed. These menus correspond to each of the datasets of the preference learning system. The items of the menu are ranked using the estimations produced by the learning system. Therefore, the preferred activities appear first so the user can easily select their favourite activities.

**Fig. 6:**
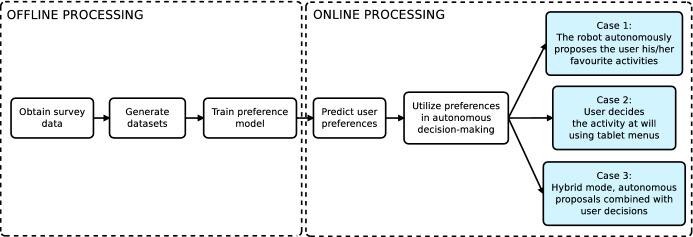
Flow diagram depicting the full process for predicting user preferences and how the autonomous decision-making system use them to provide personalized activities to different users

**Fig. 7:**
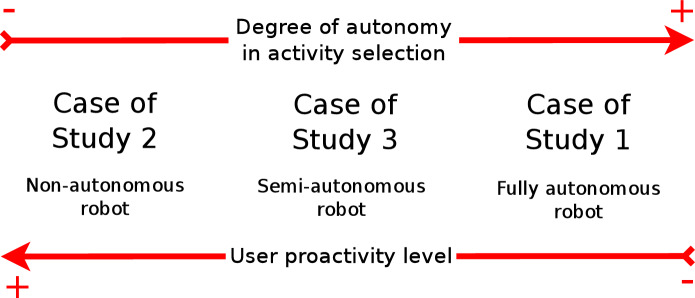
Robot autonomy level in activity selection depending on the Case of Study. A non-autonomous robot (Case of study 1) leaves decisions in the user’s hands. The selections produce using menus displayed on the touch screen. The semi-autonomous mode combined user selections with autonomous decisions of the robot using the preference learning system. Finally, a fully autonomous robot always decides the entertainment activity to execute using the preference learning system. The decision-making system selects the operating mode depending on the proactivity level of the user

Figure 6 shows the entire process of predicting user preferences and how the robot uses them to online adapt its activity selection. The above-mentioned three steps can be grouped into two phases, offline preprocessing and online processing. The first phase includes the retrieval of the survey data, the generation of the datasets used for training the preference learning model (both detailed in Sect. 5.1), and the training process itself. The second stage runs in the robot while it is interacting with the user. Starting from the trained model, when the robot interacts with a particular user, it predicts the preferences and uses them in the decision-making process Fig. 7.

### Decision-making using preference prediction in Mini

The system we present in this work provides a hierarchical user preferences prediction used by the social robot Mini in its decision-making process. This study focuses on the user’s entertainment, improving the activity selection with a robust preference estimation. The entertainment activities are organised under categories and subcategories, as depicted in Fig. 4. As stated before, the categories are *Games*, *Multimedia*, and *Information*. Games and Information categories are directly tied to a set of activities, not containing any subcategory.

The Multimedia category divides into Videos, Photos, Audiobooks, Sayings, Jokes, and Music subcategories. Each subcategory contains a group of activities at the lowest level of Fig. 4. The preference learning system predicts, for each user, different rankings about their preferences for these activities using the eight datasets. The LRF generates these rankings following the methodology explained in Sect. 3. The learning system assigns a score to each item that is used for producing the ranking. The ranking locates items with the highest score on top of the ranking. Then, the decision-making system works in three modes depending on the level of proactivity of the user, as Fig. () shows.

- The first mode activates when the user is not proactive. The robot autonomously starts the interaction with the user selecting the activity that the robot believes the user will prefer. This selection uses the preferences predicted by the preference learning model and the Boltzmann probability distribution. The hierarchical decision occurs in three steps. First, the decision-making system selects the most appropriate category, then the subcategory (if the category is Multimedia), and finally, the entertaining activity executed. Therefore, options with similar scores will present similar selection probabilities. The options with higher scores are more likely to be selected. Section 6.2.1 describes a real case of study (Case 1: A fully autonomous robot) showing how the decision-making system works in this mode.
- The second mode consists of the robot giving the initiative to the user so (s)he can decide which entertaining activity to execute. This mode activates when the robot encounters a very proactive user. The user selects the category, subcategory and entertaining activity using the tablet menus shown in Fig. 5. The items of each menu are ranked using the estimations of the preferences of each user. Preferred activities appear on top of the other to facilitate their selection. Section 6.2.2 presents a complete case of study (Case 2: A robot that gives the initiative to the user), with accurate user data to describe in more detail how this operation mode works. It is worth mentioning that despite most of the decisions are in the user’s hands, sometimes it can autonomously make partial decisions if necessary.
- The third mode combines modes one and two. This mode activates with users with moderate proactivity. In this mode, the robot combines autonomous decisions with giving the selection to the user. Thus, we allow the users to explore different alternatives from the repertoire of activities rather than leaving the entire decision in the robot’s hands. Using this method, we avoid the robot making similar decisions. Section 6.2.3 contains a detailed case of study (Case 3: A semi-autonomous robot) showing the operation of this model.

## Experiment set-up and evaluation

This section describes how to endow the social robot Mini with preference prediction for personalising its entertainment activity selection. In the first place, we present the process of building the datasets used for estimating the preferences from data retrieved using an online survey. Then, we finally explain how both LR algorithms have been compared to select the ones with the best performance. Finally, we describe the human–robot interaction experiment to validate whether the users prefer a personalised activity selection instead of a random one.

### Building datasets from surveyed data

The purpose of this work aims at predicting user preferences and use them to achieve a personalise activity selection. Like similar studies (Olsson and Salo 2011; Bouza and Bernstein 2014), we opted for spreading an online survey for building the datasets used to train the learning model. We selected a set of questions to gather socio-demographical data, habits and interests of participants. These questions are mainly related to technological issues and their preferences about the entertainment activities that Mini has. We included two additional questions about the users’ intention to socialise with other peers and whether they use electronic devices as entertainment platforms. The list of socio-demographical features obtained from the participants of the online survey was: genre, age, nationality, educational level, current occupation, number of people in their current residence, kind of place of residence, and physical exercise frequency in the week. We also asked users about habits like their use of electronic devices for entertainment and their proactivity level. Besides, we asked about their interests in sport, videogames, TV series and films, reading, photography, videos, purchases, social networks, browsing the internet, searching for information, music, and cooking, where the user had to indicate whether or not (s)he liked each category. Combining socio-demographic, habits, and interests features, each user’s input vector of features contains 21 attributes. We discarded nationality since most participants were Spaniards.

To define the labelled output of the dataset, the survey requested users to rate how much they like the entertainment activities of the robot. Ratings ranged from $0 \; to \; 5$, meaning 0 absolute dislike, and 5 total like. As stated before, robot activities are grouped in 3 categories: *Games, Multimedia* and *Information*. In *Games* category, users were requested to rate their preferences towards *Classic*, *Calculus*, and *Quiz* games. In the *Information* category, participants rated *Breaking news, National news, International news, Opinion articles, Sport news*, and *Weather forecast*. Finally, the *Multimedia* category rated *Jokes*, *Sayings*, and different types of *Music* (*English pop, English rock, Spanish pop, Spanish rock, Classical, Latin*, and *Flamenco*), *Audiobooks* (*Classics, Children’s Tales*, and *Historical events*), *Photos* (*Landscapes, Funny moments, Monuments*, and *Animals*) and *Videos* (*Sports, Cooking, Comedy*, and *Film trailers*). Each category and subcategory defines a dataset that contains a particular ranking for each user. Using the ratings provided by each participant, activities with higher ratings are ranked on top. Rating ties were randomly broken. Using the activities classification in categories and subcategories and the rankings, we built 8 datasets. The first dataset ranked the categories (Games, Multimedia, and Information). The other datasets ranked the activities in the categories *Games, Information*, and *Multimedia* and the subcategories *Photos, Videos, Audiobooks*, and *Music*. Moreover, we built 2 additional datasets, *Entertainment* and *Multimedia*. This dataset organisation allows the decision-making system to select a category hierarchically, then a subcategory, and finally an entertainment activity that will be executed. Thus, all datasets are necessary to personalise the activity selection since the ranking they contain indicates which categories, subcategories, and entertainment activities each user likes the most and will be selected more often.

We received 473 replies to the online survey, which led to 471 input instances since three replies were not valid due to inconsistencies in the features (for example, answering being 8 years old and a qualified worker). The participants were 217 men, 254 women aged $\mu =29.52, \sigma =12.64$. $96\%$ of them were Spaniards. Valid replies defined the input instances and labelled output of the 8 datasets utilised by the preference learning algorithms during the training and validation of the model. When the robot interacts with new users, their features have been obtained using a questionnaire containing the questions listed in Appendix A. We are currently working on including these questions in the robot to obtain the users’ features using human–robot interaction. Then, using these features, the preference learning system predicts the user preferences organising them in three decision levels that are used by the decision-making system like Sect. 4.3 describes.

### Ranking correlation metrics

Preference learning predictions are aggregated rankings that represent the preferences of an instance . Preference learning methods use Kendall’s $\tau$-b (Kendall 1945) and Spearman’s $\rho$ (Spearman 1961) nonparametric correlations to evaluate the estimation yielded the model. Both metrics range from $[-1,1]$. Values close to 1 represent a solid positive rank correlation (perfect estimation), while correlations close to $-1$ indicate a strong negative correlation (ranking reversed). Drawing on (Corder and Foreman 2011; Schober et al. 2018), we will consider positive, strong rank relation values above $+0.7$, moderate correlation values between $+0.3$ and $+0.7$, and weak otherwise.

**Kendall’s** $\varvec{\tau }$-**b** (Kendall 1945) developed this nonparametric measure of correlation between two rankings $\pi$ and $\pi '$, like Eq. 3 shows. It relies on the number of concordant ($n_{c}$) and discordant ($n_{d}$) pairs of the ranking, considering the number of ties in the predicted ranking $\pi$ ($t_{i}$) and in the real ranking $\pi '$ ($u_{j}$).

3 $$\begin{aligned} \tau = \frac{n_{c}-n_{d}}{\sqrt{(n_{c}+n_{d}+t_{i})*(n_{c}+n_{d}+u_{j}))}} \end{aligned}$$

**Spearman’s** $\varvec{\rho }$ (Spearman 1961) rank-order correlation is a nonparametric measure of the monotonicity between the correspondence of two rankings $\pi$ and $\pi '$. Unlike other correlation metrics such as Pearson’s coefficient (Gao et al. 2016), Spearman’s $\rho$ does not assume a normal distribution of the data, which makes it more suitable to our self-built dataset. As Eq. 4 represents, it depends on the number of pairs *N* available in the ranking and the distance $D(\pi , \pi ')$ about the positioning of each label in both rankings.

4 $$\begin{aligned} \rho = 1 -\frac{6\sum _{i=1}^{N} D_{i}(\pi , \pi ')^{2}}{N(N^{2}-1)} \end{aligned}$$

### Evaluation of the ranking algorithms

Ranking by Pairwise Comparison and Top Label as Class were evaluated in terms of the average prediction accuracy of the random forest integrated into them, the Kendall $\tau$-b and Spearman $\rho$ ranking correlation metrics, and the training time per cross-validation iteration during training and validation. The three first metrics represent the model’s accuracy in predicting rankings, whereas the training time is essential to determine whether the system can run in real time in the robot as one of the future goals will be to retrain the system online.

The first step in this process was to find the hyperparameters of both algorithms that produce better results in each case. Ranking by Pairwise Comparison, Top Label as Class and Random Forest (the classifier) have multiple parameters. We opted for using the node splitting criteria (Information gain (Quinlan 1996) or Gini impurity (Steinberg and Colla 2009)), the number of trees of the forest ($n_{trees}$), the maximum depth of the tree ($m_{depth}$), the minimum number of samples in a node to produce a splitting ($n_{samples}$) and the minimum number of samples in a node to directly become a leaf of the tree ($n_{leaf}$) since they are the most representative ones in terms of its influence over the performance of both algorithms. Hyperparameter optimisation was carried out using a grid search method (Syarif et al. 2016), so we defined the optimisation ranges of each parameter according to Table 1.

**Table 1:** Range of values used for each hyperparameters during grid-search optimization

Hyperparameter	Grid-search values
Splitting criteria	Gini impurity, Information gain
$n_{trees}$	[10, 20, 30...150]
$m_{depth}$	[2, 3, 4, 5, 6]
$n_{samples}$	[5, 10, 15, 25]
$n_{leaf}$	[10, 15, 20, 25]

### Human–robot interaction assessment

Once we found the best algorithm for our application, it was necessary to validate the preference learning system. Thus, we tested whether selecting entertainment activities using the user’s preferences was perceived more positively than selecting activities at random. The experiment consisted of 22 participants (13 women, 9 men aged $\mu =41.09$, $\sigma =20.57$, all Spaniards) interacting with the social robot Mini during short-term interactions. Before interacting with the robot, all participants filled the questionnaire shown in Appendix A to obtain their input features that were necessary for predicting their preferences. Then, each participant was randomly assigned to one of the two conditions tested: Interacting with a robot that selects the activities using the preference learning system presented in this work (Condition 1) and interacting with a robot that randomly decides the activities to execute (Condition 2). Hence, the participants were equally distributed in both conditions (11 participants in each condition). Independently of the condition of participants, during the interaction, they realised three consecutive activities selected from the repertoire shown in Fig. 4.

At the beginning of the experiment, the robot started with a short introduction about its dynamics. Then, the participant had to execute the three activities selected by the robot. On average, the duration of each session lasted around ten minutes, considering the duration of each activity around three minutes each plus one minute of introduction. We included two questionnaires for the evaluation that the participants had to complete after interacting with the robot. The first questionnaire contained six ad hoc questions about the perception of the robot’s personalised activity selection (*Personalisation perceived*). We decided to include customised questions considering previous similar works in the evaluation of social robots (Churamani et al. 2017; Tozadore et al. 2018). The participant answered the six questions using a 5-point Likert scale where one means strongly disagree and five strongly agree. The aggregated results of the six questions reported the attribute Personalisation perceived. The six ad hoc questions are listed below.

- **Q1: **How appropriate did you find the first activity selected by the robot according to your preferences?
- **Q2: **How appropriate did you find the second activity selected by the robot according to your preferences?
- **Q3: **How appropriate did you find the third activity selected by the robot according to your preferences?
- **Q4: **In general, do your think the activities proposed by the robot are adequate to you?
- **Q5: **Have you noticed that the robot knows information about yourself?
- **Q6: **Have you noticed that the robot behaviour adapts to yourself?

Following the ad hoc questions, the second questionnaire consisted of the well-known Godspeed questionnaire (Bartneck et al. 2008), used for evaluating different attributes of social robots. The questionnaire evaluates the robot in five different general attributes: Anthropomorphism (A), Animacy (A), Likeability (L), Intelligence perceived (IP), and Security perceived (SP). Each attribute has three to five items that use a 5-point Likert scale allowing the participant to indicate how (s)he perceives the robot between two opposite terms (e.g. artificial vs lifelike). Since the public aim of the study was Spaniards, we used the official translation provided in (Weiss and Bartneck 2015).

In this evaluation, we expected to obtain significant statistical differences between both conditions tested in the personalisation perceived and some of the categories of the Godspeed questionnaire. The attributes Likeability (L), Intelligence perceived (IP), and to a lesser extent Animacy (AN) are those more related to adaptive and personalised activity selection, so we believed that participants of Condition 1 would perceive the robot exhibiting a personalised activity selection prominent in these attributes. However, we did not expect to obtain differences in the Anthropomorphism (A) and the Security Perceived (SP) attributes as both are related to the robot’s physical features without any influence on personalised activity selection.

## Results

**Fig. 8:**
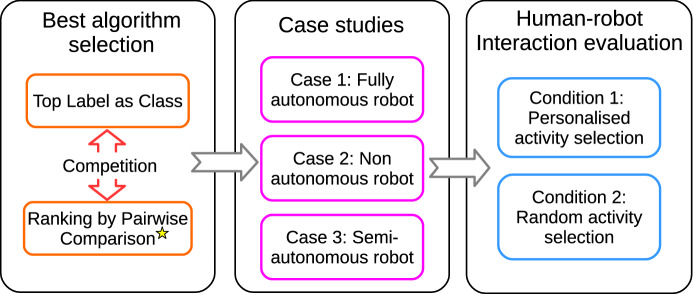
Results carried out to test the operability of the preference learning system in the social robot Mini. First, we compare Top Label as Class and Ranking by Pairwise Comparison to obtain the algorithm with the best performance in our application. Then, we present three case studies about the operation of the system when interacting with new users with different proactivity levels. Finally, we validate the system in real human–robot interactions to assess whether the personalised selection of entertainment activities is preferred above a random activity selection

The following section presents the results obtained in selecting the best preference learning algorithm regarding the hyperparameters optimisation, the prediction accuracy of random forest and ranking metrics. Once we selected the best algorithm, we describe three case studies showing the operation modes of the decision-making system during the recommending the preferred entertainment activities of the robot. Finally, we conclude by presenting the results of the preference learning system performance in real human–robot interactions. Figure 8 shows the process of validating the preference learning system presented in this manuscript.

### Algorithm selection

The selection of the LRF algorithm that produces the best outcomes in our multi-dataset hierarchical learning system led to comparing Ranking by Pairwise Comparison and Top Label as Class, two outstanding methods with different features. Next, we show the comparison results and select the LRF method more appropriate for our model.

#### Optimising hyperparameters

**Fig. 9:**
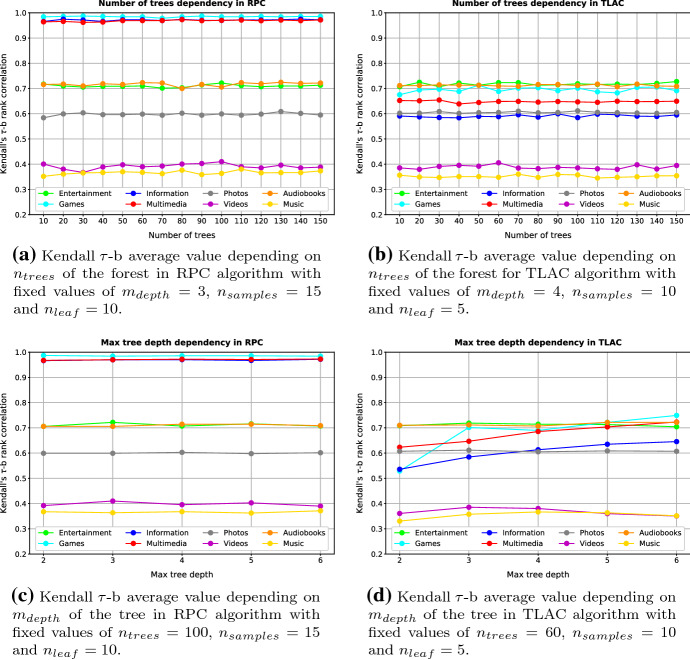
Representation of $n_{trees}$ and $m_{depth}$ hyperparameters optimization on each dataset considering the set of hyperparameter that provided the best results in each trial

Figure 9 represents Kendall’s $\tau$-b ranking correlation according to the number of trees per forest $n_{trees}$ and the maximum depth of each tree $m_{depth}$ using Gini impurity as splitting criteria for Ranking by Pairwise Comparison and Top Label as Class algorithms. Note that Kendall’s $\tau$-b metric is represented in these graphs from 0.2 to 1.0, although its output range is $[-1, 1]$ since no value was below 0.2.

Using the results provided by Fig. 9, it is possible to assure that the best set of hyperparameters for Ranking by Pairwise Comparison is $n_{samples}=15$, $n_{leaf}=10$, $n_{tress}=100$, and $m_{depth}=3$. Regarding Top Label as Class, the best performance is obtained with $n_{samples}=10$, $n_{leaf}=5$, $n_{tress}=60$, and $m_{depth}=4$. Thus, the results presented in the following sections use this configurations for each algorithm.

#### Prediction accuracy

Table 2a shows the prediction accuracy mean and standard deviation (in brackets) values obtained during a tenfold cross-validation testing phase of each dataset, using both algorithms and both splitting criteria. This metric is a reasonable estimation of the performance of the ranking since it is the preliminary step to rank aggregation techniques.

Results show how Ranking by Pairwise Comparison produces better classifications than Top Label as Class for all cases. Moreover, Gini impurity usually presents better results than Information gain. Focusing on the numerical results of the classification, the high accuracies in the Games, Multimedia and Information datasets are remarkable, all above $98\%$ of success. Considering Entertainment, Photos and Audiobooks datasets, the classification accuracy is good in Ranking by Pairwise Comparison (all above $80\%$) but drops when classifying with Top-Label as Class. Finally, in Videos and Music datasets, the classification accuracy is around $70\%$ in both cases for Ranking by Pairwise Comparison algorithm, but diminishes for Top Label as Class, especially in the Music dataset ($\sim 38\%$). Note that the differences in the classification accuracies of each dataset are due to different causes. First, the number of items in each ranking reduces the accuracy as the complexity of the model increases. Second, the definition and structure of the dataset may contain inconsistencies causing a reduction in the prediction accuracy.

#### Training time

Table 2b represents the average training time for both algorithms during training and validation stages. At first glance, it is worth mentioning how Top Label as Class requires less computational time than Ranking by Pairwise Comparison, especially for those cases where the number of ranking labels is high (Music, Information and Multimedia datasets). These results support one of the hypotheses previously stated in this manuscript, Top Label as Class requires less training time than Ranking by Pairwise Comparison. This variation is due to the ranking splitting and aggregation processes performed before and after the random forest classification. Top Label as Class only trains and evaluates one model for each dataset, independently of the number of available labels to rank in such dataset.

Ranking by Pairwise Comparison requires additional computational time as it trains $m(m-1)/2$ models per dataset, where *m* is the number of items to rank. Although Top Label as Class trains one model per dataset, the number of items to rank also affects the training time, but to a lesser extent. Finally, the comparison of Gini impurity with Information gain presents very similar values, not showing any significant difference.

**Table 2:** Numerical results in terms of prediction accuracy of the random forest classifier, training time, Kendall’s $\tau$-b and Spearman’s $\rho$ ranking correlation metrics for Ranking by Pairwise Comparison (RPC) and Top Label as Class (TLAC) algorithms using Gini impurity and Information gain as splitting criteria on the decision tree. **Bolded numbers** indicate the best algorithm for each dataset and splitting criteria assessed in the comparison

Dataset	Algorithm				
	RPC		TLAC		
	Gini impurity	Information gain	Gini impurity	Information gain	
(a) Mean (std) average values for internal random forest classifier accuracy obtained using optimal hyperparameters in tenfold cross-validation.					
Entertainment	**.8630 (.0227)**	.8630 (.0308)	.7326 (.0583)	.7347 (.0788)	
Games	.9934 (.0068)	**.9942 (.0054)**	.9804 (.0180)	.9934 (.0099)	
Multimedia	.9862 (.0047)	**.9872 (.0044)**	.9217 (.0325)	.9500 (.0377)	
Information	**.9886 (.0053)**	.9881 (.0044)	.9304 (.0474)	.9000 (.0468)	
Photos	**.8057 (.0137)**	.8050 (.0283)	.7456 (.0937)	.7500 (.0668)	
Videos	**.7065 (.0191)**	.7050 (.0222)	.6239 (.0558)	.6282 (.0655)	
Audiobooks	.8666 (.0269)	.8681 (.0252)	.8760 (.0292)	**.8826 (.0403)**	
Music	**.6962 (.0226)**	.6928 (.0154)	.3847 (.0550)	.3739 (.0946)	

Dataset	N Labels	Algorithm			
		RPC		TLAC	
		Gini Impurity	Information Gain	Gini Impurity	Information Gain
(b) Mean training time values per each cross-validation iteration (training and validation), which depend on the number of labels of each ranking using optimal hyperparameters.					
Entertainment	3	3.8511	3.8414	2.4019	**2.2970**
Games	3	4.1858	4.0515	**2.0990**	2.1385
Multimedia	6	18.3387	17.8568	**3.3404**	3.3899
Information	6	18.5684	18.4749	4.4734	**3.8260**
Photos	4	7.2872	7.2957	2.8319	**2.6490**
Videos	4	7.9843	8.0638	2.5251	**2.4801**
Audiobooks	3	3.7682	3.9130	2.3731	**2.3180**
Music	7	26.7077	26.6414	5.5840	**5.0993**

Dataset	Algorithm				
	RPC		TLAC		
	Gini Impurity	Information Gain	Gini Impurity	Information Gain	
(c) Mean (std) Kendall’s $\tau$-b average values for tenfold cross-validation obtained for optimal hyperparameters.					
Entertainment	**.7260 (.0455)**	.7260 (.0616)	.7246 (.0349)	.7246 (.0685)	
Games	.9869 (.0136)	**.9884 (.01084)**	.7637 (.0954)	.8362 (.0534)	
Multimedia	.9721 (.0113)	**.9753 (.0127)**	.7315 (.0286)	.7292 (.0223)	
Information	**.9762 (.0112)**	.9744 (.0090)	.6530 (.0286)	.6997 (.0314)	
Photos	**.6115 (.0275)**	.6113 (.0584)	.6088 (.0897)	.6037 (.0471)	
Videos	.4007 (.0412)	**.4094 (.0538)**	.4057 (.0376)	.4014 (.0669)	
Audiobooks	.7333 (.0539)	**.7362 (.0505)**	.7289 (.0439)	.7318 (.0481)	
Music	**.3805 (.0460)**	.3799 (.0284)	.3726 (.0427)	.3788 (.0338)	

Dataset	Algorithm				
	RPC		TLAC		
	Gini Impurity	Information Gain	Gini Impurity	Information Gain	
(d) Mean (std) Spearmans’s $\rho$ average values for tenfold cross-validation using optimal hyperparameters.					
Entertainment	.7576 (.0462)	**.7608 (.0631)**	.7554 (.0315)	.7521 (.0660)	
Games	.9902 (.0102)	**.9913 (.0081)**	.8206 (.0711)	.8739 (.0438)	
Multimedia	.9831 (.0069)	**.9849 (.0090)**	.8108 (.0290)	.8126 (.0221)	
Information	**.9853 (.0081)**	.9848 (.0062)	.7636 (.0248)	.7983 (.0286)	
Photos	.6765 (.0285)	.6765 (.0641)	**.6782 (.0881)**	.6734 (.0468)	
Videos	.4465 (.0555)	.4486 (.0590)	**.4630 (.0328)**	.4578 (.0681)	
Audiobooks	**.7717 (.0500)**	.7706 (.0450)	.7684 (.0434)	.7706 (.0436)	
Music	.4781 (.0550)	.4754 (.0330)	.4760 (.0563)	**.4835 (.0333)**	

#### Kendall $\tau$-b rank correlation

Table 2c shows Kendall’s $\tau$-b correlation values for each dataset in terms of its mean and standard deviation values (in brackets). According to the results, Ranking by Pairwise Comparison performs substantially better than Top Label as Class in the Games, Multimedia, and Information datasets. In the other datasets, Kendall’s $\tau$-b values are very similar. However, Ranking by Pairwise Comparison outperforms Top Label as Class in all datasets. Gini impurity and Information gain do not present significant discrepancies, which means that the results are independent of the splitting criteria used intrinsically on decision tree classifiers.

Concentrating on Kendall’s $\tau$-b correlation and not comparing both algorithms, the preference learning setting provides promising results in terms of rank correlation. In Games, Multimedia, and Information datasets, Kendall’s $\tau$-b values are above 0.97, which means a very robust rank correlation at the testing phase. Results are excellent in the Entertainment and Photos datasets, as rank correlation is strong, above 0.7. Focusing on the Photos dataset, a high, moderate rank correlation is obtained (0.61). Videos and Music datasets present worse rankings in rank correlation (around 0.4), classified as low, moderate rank correlation. Remember that positive correlations above $+0.7$ are strong, values between $+0.3-+0.7$ moderate, and weak otherwise (Corder and Foreman 2011; Schober et al. 2018). Also, note that the datasets with worse results correspond to those with lower classification accuracies and with a higher number of items to rank. This issue suggests that estimations are strongly dependent on the length of the ranking.

#### Spearman $\rho$ rank correlation

Table 2d shows the results obtained for Spearman’s $\rho$ rank correlation. It shows the mean and standard deviation values (in brackets) for each LRF algorithm and splitting criteria using optimal hyperparameters. The use of this correlation metric supports the results provided by Kendall’s $\tau$-b. Despite this enormous similarity, the discrepancies between Ranking by Pairwise Comparison and Top Label as Class found in Table 2c for some datasets are less notable in Table 2d. This issue is probably because Kendall’s correlation metric considers both concordant and discordant pairs in the rankings being more severe than Spearman’s correlation, being more thorough than Kendall’s $\tau$-b in comparing real and estimated rankings.

#### Best algorithm selection

Considering the results obtained from the comparison of Ranking by Pairwise Comparison and Top Label as Class, we opted for selecting Ranking by Pairwise Comparison since it generally provided better estimations. As results in Table 2 show, Ranking by Pairwise Comparison using Gini impurity subtly outperforms the other alternatives. However, the differences between Gini impurity and Information gain are almost undetectable. Contrary to our initial believe, this fact means that the splitting criteria is not a critical hyperparameter of our learning system.

As we expected, Ranking by Pairwise Comparison provides substantially better results for the Games and Multimedia datasets than Top Label as Class, probably because their dataset definition ballast the replacement of the top-label carried out by Top Label as Class. Although in the other datasets Ranking by Pairwise Comparison still outperforms Top Label as Class, in these cases the differences are minimal. Remember that these results arise from the hyperparameter optimisation method described in Fig. 9.

### Case studies

The following section presents three case studies describing Mini’s autonomous entertainment activity selection using the user preferences and the level of proactivity. These cases correspond to the three operation modes of the decision-making system explained in Sect. 4.3. Case 1 compares the autonomous activity selection for two users with different features but a low proactivity level. Case 2 shows that, for very proactive users, the robot opts to leave the decision in the user’s hands. Finally, Case 3 combines both previous approaches for users with a moderate level of proactivity.

**Table 3:** Input vector representing the 21 features denoting the profiling of the three users used in the case studies where we describe the three operation modes of the decision-making system according to their level of proactivity

Feature	User 1	User 2	User 3
Genre	Female	Male	Male
Age	50	44	27
Education	Primary School	Medium level Vocational Training	Bachelors
Occupation	Unemployed	Skilled worker	Student
Coexistence	Familiar	Familiar	Shared house
Place of residence	Village/ Countryside	Small city	Big city
Physical activity	Less than once a week	Less than once a week	1-3 times a week
Habits Electronic devices	Yes	No	Yes
Proactivity level	1	1	5
Interest in Sport	No	No	Yes
Interest in Videogames	No	No	Yes
Interest in TV series and films	Yes	Yes	Yes
Interest in Reading	No	No	No
Interest in Photography	No	No	No
Interest in Videos	No	No	Yes
Interest in Purchases	No	No	No
Interest in Social Networks	Yes	No	Yes
Interest in Browsing the Internet	Yes	No	Yes
Interest in Searching Information	No	No	Yes
Interests in Music	Yes	No	No
Interest in Cooking	No	No	No

#### Case 1: a fully autonomous robot

**Fig. 10:**
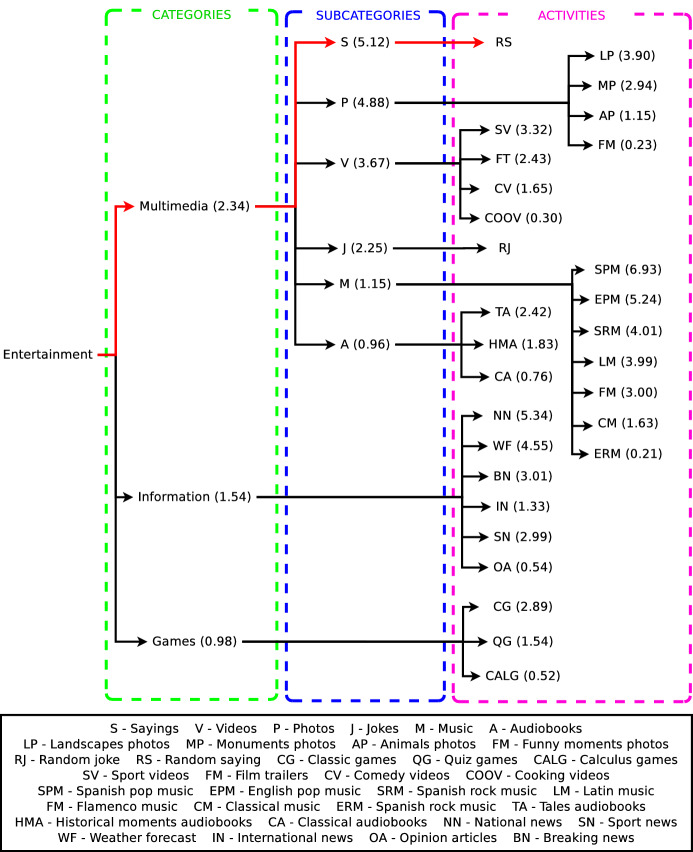
Predicted decision tree for User 1 showing how each category, subcategory, and activity rank in the hierarchy according to the score (in brackets) estimated by the preference learning system. Highlighted in red, the path that represents the preferred category, subcategory, and entertainment activity for the user. In this case, the category is Multimedia, the subcategory is Sayings, and the activity is playing a random saying

**Fig. 11:**
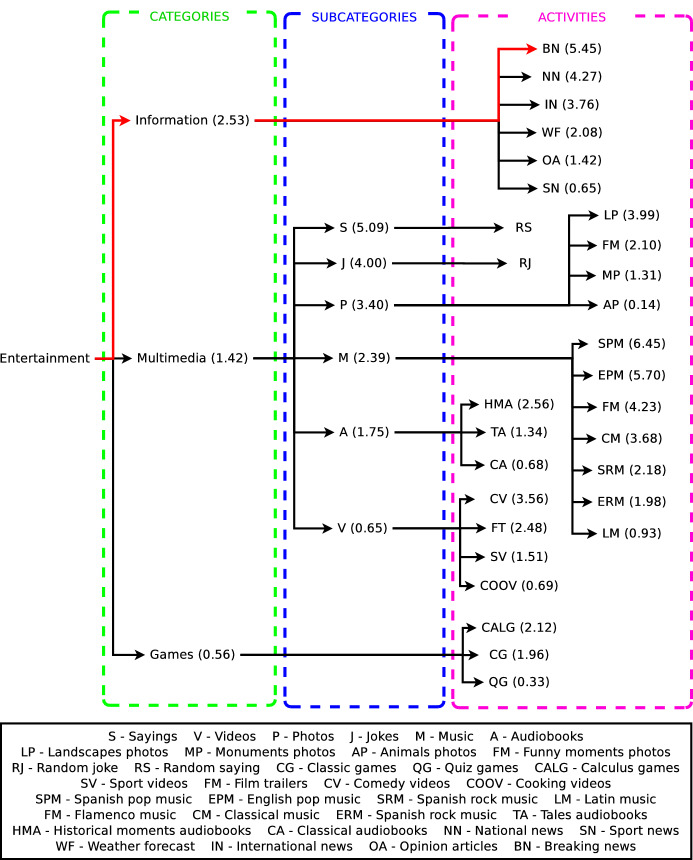
Predicted decision tree for User 2 showing how each category, subcategory, and entertainment activity rank inside the hierarchy according the score of each item. Highlighted in red, the path indicating the activity breaking news (BN) contained in the Information category that the user likes the most

This case of study shows how the preference learning system presented in this work produces different preference estimations for two different users. Thus, the decision made by the decision-making system of the robot adapts to the estimated preferences of each user, producing a personalised interaction. Table 3 shows the input features of User 1 and User 2 given to the preference learning model to estimate their preferences towards the activities of the robot in hierarchical levels. Considering their input vector of features, our preference learning model predicts a ranking of labels for each dataset, hierarchically represented as decision trees. Figure 10 shows the decision tree for User 1 and Fig. 11 for User 2. Both trees organise the activities of the robot in categories and subcategories, like Fig. 4 shows, but following a ranking format. Due to the length in some entertainment activities names, a legend supports the understanding of the figures. In Figs. 10 and 11, each category, subcategory, and entertainment activity in the tree has a score in brackets next to the name of the item. These scores are the preferences of the user that originate the rankings. Thus, for example, the ranking of categories for User 1 is Multimedia (2.34) > Information (1.54) > Games (0.98). This method extrapolates to the other rankings generated for each of the eight datasets shaping the preference learning. Since both users in this example have a low proactivity level (see Fig. 3), the decision-making system of the robot will work in a fully autonomous mode. Thus, the robot will always make autonomous decisions selecting the entertainment activity to execute considering the predictions of the preference learning system. It is worth mentioning that each item’s scores depend on the ranking contained in each dataset and not on the overall architecture. Besides, scores range from 0 to 5, depending on the users’ ratings in the survey.

The predictions for User 1 lead the decision-making to select more often specific entertainment activities. These selections depend on the temperature values set in the Boltzmann Eq. 2. Looking at Fig. 10, the red pathway represents the preferred category, subcategory, and entertainment activity for User 1 predicted by the preference learning model. An example of the operation of the decision-making system considering the features of User 1 if the Temperature of the Boltzmann equation values 0.1, starts with the selection of the preferred activity of User 1. Thus, the robot will select the Multimedia category, then the Sayings (S) subcategory, and finally, it will execute a random saying activity (RS). Note that the random saying activity does not have a score since it is the only activity inside the Sayings (S) subcategory. Otherwise, if the Temperature in the Boltzmann equation values 100 units, the estimated preferences are omitted, and the category, subcategory, and entertainment activity are selected randomly. If the temperature parameter in the Boltzmann equation is between $0.1-100$, the selection of the preferred activity of the user is not assured. As the temperature value increases, selections are more random since the likelihood assigned by the Boltzmann equation to each item in the ranking is more similar. The temperature value is beneficial to introduce a degree of randomness in the activity selection, avoiding the decision-making system always selecting top-ranked items.

Considering the features of User 2 in Table 3 and the decision tree estimated by the preference learning system in Fig. 11, the red path denotes the preferred activity of User 2. When deciding which entertainment activity to execute, if the temperature parameter in the Boltzmann equation is set to its lower value of 0.1 units, Mini will perform the selection indicated in red in Fig. 11. Thus, an example of the operation of the decision-making in this operation model starts by selecting the category Information in the first place because it is the one with the highest score among all categories. Since the Information category does not contain any subcategory, the following selection will directly be the entertainment activity contained in the Information category. In this case, the entertainment activity breaking news (BN) is the preferred activity as it has the highest score from all the other activities (national news (NN), international news (IN), weather forecast (WF), opinion articles (OA), and sports news (SN) in this order). If the temperature value is close to 100, the selection likelihood of each item will be homogenised, leading to a random category, subcategory, and activity selection. For this reason, including a variable temperature value allows the robot to explore new activities instead of repeating the selection of the preferred one.

This autonomous operation mode presents the advantage of proactively start the interaction with inexperienced users or those with interaction limitations. Thus, the robot fosters interacting with the user engaging him/her with entertainment activities. On the other hand, always selecting the activities without the intervention of the user may lead to repeat the preferred activities provoking the user’s fatigue. Besides, the estimations produced by the preference learning system may not be the real preferences of the user, so this method would require a complementary adaptive refinement of the initial predictions with the interaction.

#### Case 2: a robot that gives the initiative to the user

This case of study shows the second operation mode of the decision-making system. This mode activates when the robot interacts with users with a high level of proactivity. In this mode, the robot guides the user during the entertainment activity selection process, but the user decides which activity the robot will finally execute. Like in the previous mode, the activity selection follows a three-level hierarchical path. First, the user has to select the category, then the category, and finally the entertainment activity that (s)he prefers. A sequence of tablet menus is displayed on the touch screen to assist the user in selecting and letting him/her know about what activities it has. Figure 5 shows the organisation of the entertainment activities of the robot in tablet menus. In operation mode, the scores of each item in the ranking are not used because the decision-making system does not work autonomously. However, the rankings generated by the preference learning model serve to organise the tablet menus according to the user’s preferences. Thus, each category and subcategory that the user prefers will appear on top of the tablet menu, facilitating their selection. Although this approach does not imply the complete functionality of the Preference Learning system, it allows users to select the activities they prefer, embedding ranking predictions in the tablet menus so the favourite categories, subcategories, and activities can be easily seen when making the decision.

An example of this operation mode could be the following. Initially, the Entertainment menu will be permanently displayed, ranking the categories Games, Multimedia, and Information (Fig. 5a) according to the preferences of the user. If the user selects the Games category, a new tablet menu (Fig. 5b) will pop up displaying the gaming activities of the robot (classical, calculus, and quiz games) so the user can make its decisions. In case the Multimedia category is selected, the new menu appearing will be Multimedia (Fig. 5c), ranking the subcategories Sayings, Jokes, Videos, Photos, Music, and Audiobooks. Then, depending on the user selection, the last menu will appear on the touch screen. If the selection is Music, the different music styles (Fig. 5h) will appear ranked according to the preferences of the user. Once the music style has been selected, a song will start playing.

Contrary to the first operation mode, this method has the advantage of allowing users to select their favourite entertainment activity, so the probabilities of making an incorrect selection are substantially reduced. This method is useful for adapting the initial predictions with the interaction as users are providing positive feedback by selecting their preferred activities. However, with users that do not like the robot and refuse to interact with it, this mode can lead the user to stop the interaction since (s)he can feel that the robot behaves like a simple automaton.

#### Case 3: a semi-autonomous robot

**Fig. 12:**
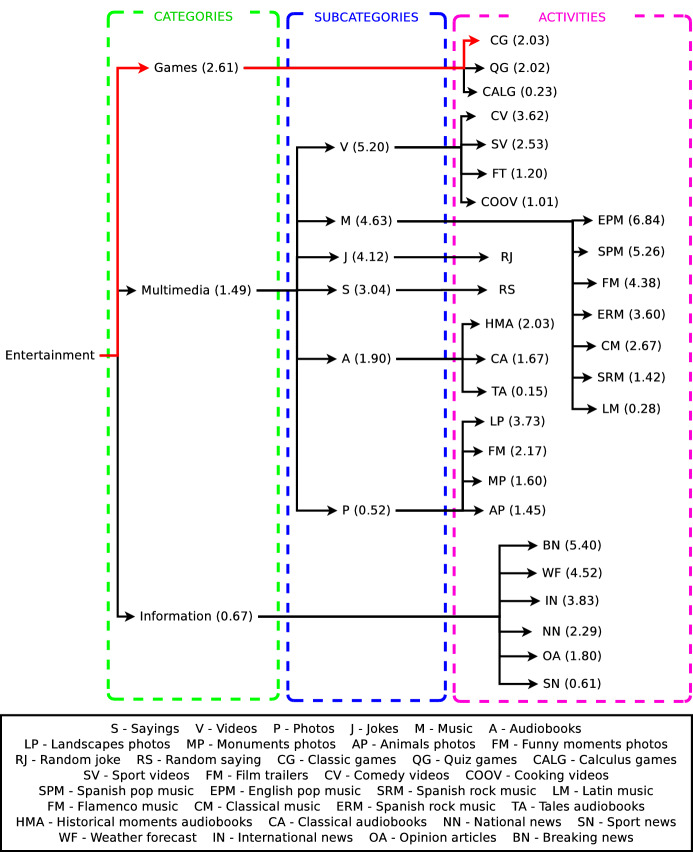
Predicted decision tree for User 3 showing how each category, subcategory, and activity rank inside the hierarchy according to its associated estimated score (in brackets). Highlighted in red, the path indicating which activity (classic games (CG)) has more possibilities to be chosen in case the robot decides the activity autonomously

The third case of study presents how the decision-making system of the robot balances autonomous decisions with giving the initiative to the user. This operation mode activates for users with a moderate proactivity level. Considering the feature of User 3 in Table 3 and the prediction tree shown in Fig. 12, the decision-making system intercalates autonomous decisions with displaying tablet menus. The probability of making autonomous decisions or displaying a tablet menu is randomised at each decision step. Thus, in this case, the probability of being autonomously decided by the robot or the user is the same. If the robot makes the decision, the preferences estimated and the Boltzmann equation are used like Sect. 6.2.1 shows. If the user makes the selection, the methodology is stated in Sect. 6.2.2. Then, the subcategory and the entertainment activity are selected following the same method. Since the robot works in a semi-autonomous mode, the subcategory and activity can be selected by the robot or the user with equal probability.

An example of operation mode that considers the rankings and scores predicted for User 3 shown in Fig. 12 could be as follows. First, the system decides if the robot or the user selects the category. If the robot autonomously selects the category, the the preferred item for User 3 according to the estimation of the preference learning algorithm is Games. Therefore, if the temperature in the Boltzmann equation is low, Games will likely be the selected option. If the temperature is high (close to 100 units), the category will be randomly selected. If we assume that the robot selects the Games category and then determines the user has to make the following decision, a tablet menu containing the activities related to games will be displayed (Fig. 5b). Using this menu, the user can select their favourite gaming activity from classical, quiz, and calculus games, displayed in that order. If the selection depends on the rankings estimated by the Preference Learning system, the red path in Fig. 12 will be selected more often since it leads to the user’s favourite activity (classic games).

Considering the case studies presented in this section about how the decision-making system of Mini works, the level of exploration (in terms of executing a broader range of different activities) depends on two factors. First, it depends on the temperature of the Boltzmann equation when the robot autonomously decides what item to select at each decision stage using estimated preferences for a particular user. Additionally, the exploration is also higher when more decisions depend on the user, as (s)he can decide the real preferences (which can be different from the estimated ones). Although allowing the user to select by (him)herself what activity the robot will execute may promote exploration, autonomous decision-making becomes especially necessary for users with interaction limitations and low proactivity.

From our point of view, this operation mode will provide the best results in the long run. We hypothesise that the combination of the robot’s autonomous decisions with user selection could reduce the fatigue of the user and increase the engagement. Besides, balancing the activity selection may drive the user to test a broader repertoire of entertainment activities, discovering their real favourite ones. A possible drawback of this method can be related to the expectations of the user. If (s)he is waiting for a menu to appear and the robot decides a different activity, the user may feel that the robot is not really adapting to him/her. For this reason, it is important that autonomous and user selection are correctly balanced to avoid these situations.

### Human–robot interaction results

**Fig. 13:**
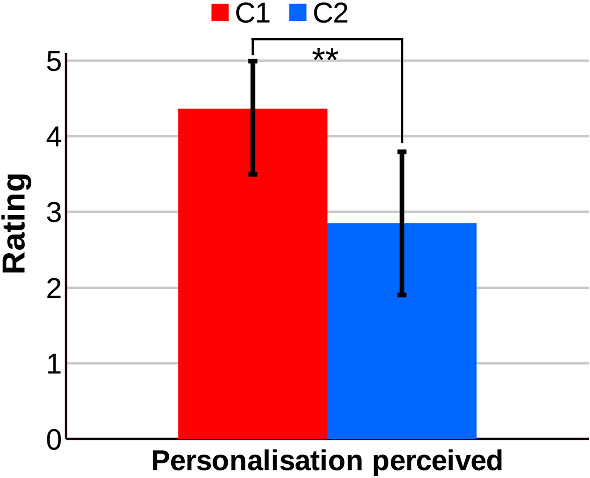
Mean and standard deviation value of the Personalisation perceived attribute for Condition 1 (C1): a robot personalising the activity selection and Condition 2 (C2): a robot selecting activities randomly. Very significant statistical differences (*p*-value from 0.01 to 0.001) are denoted with ***

The evaluation of the system during human–robot interaction provided valuable results regarding both conditions tested. Figure 13 compares the mean and standard deviation values for the attribute *Personalisation perceived* reported by the participants of Condition 1 (personalisation) and Condition 2 (general).

The statistical analysis of the Personalisation perceived, presented in Table 4, befell using the Mann–Whitney’s U (Zimmerman 1987) nonparametric statistic since the data did not follow a normal distribution and the sample is relatively small. The analysis shows significant statistical differences between both conditions ($p-value=0.003$). This fact indicates that the participants using a robot with personalised activity selection (Condition 1) found the robot more adaptive to themselves than those with random activity selection (Condition 2).

**Table 4:** Results obtained from the statistical analysis of the Personalisation perceived regarding the personalisation of the activity selection process Very significant statistical differences (p-value < 0.001) are indicated with **

Attribute	Mann-Whitney’s U	*p*-value
Personalisation perceived	16.50	.003**

**Fig. 14:**
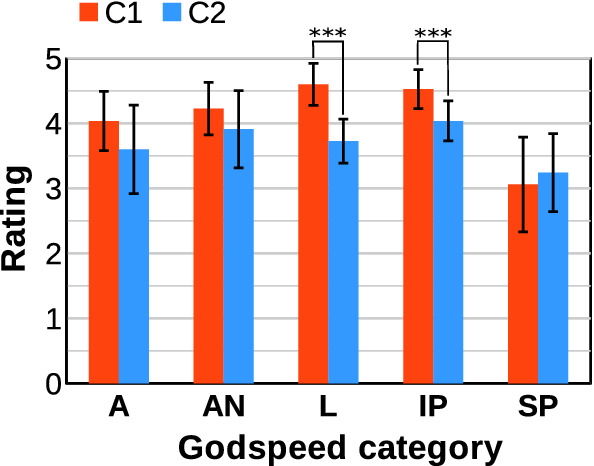
Mean and standard deviation values in the statistical analysis of the Godspeed results considering each category and condition tested (Condition 1 (C1): a robot personalising the activity selection and Condition 2 (C2): a robot selecting activities randomly). Anthropomorphism (A), Animacy (A), Likeability (L), Intelligence perceived (IP), and Security perceived (SP). Significant statistical differences (*p*-value from 0.05 to 0.01) are denoted with *, very significant (*p*-value from 0.01 to 0.001) with **, and extremely significant (*p*-value$<0.001$) with ***

The analysis of the results related to the Godspeed questionnaire lead to the following conclusions. As Fig. 14 shows, the participants that interacted with a robot that personalised the activity selection (Condition 1) generally rated the robot higher than those participants that interacted with a robot that randomly decided the activities to execute (Condition 2). The only category that received a lower rating by participants in Condition 1 was the Security Perceived (SP). However, the ratings in both conditions regarding this category were very similar.

The statistical analysis of the data also provided valuable results in the comparison of both conditions. Since the data retrieved in the evaluation followed a normal distribution, we could apply parametric statistics. In our experiment, we have two independent samples (Condition 1 and Condition 2). For this reason, the most appropriate statistical analysis to investigate whether there exists a significant difference between them is the Student’s T-test (Efron 1969). Table 5 shows the statistical analysis for each category in the Godspeed questionnaire; each category contains several degrees of freedom (DoF) that equally contribute to the evaluation of the category. Results show significant statistical differences for the Student’s T-test in the categories Likeability (L) (*p*-value$<0.001$) and Intelligence Perceived (IP) (*p*-value=0.01) and no differences in the categories Anthropomorphism (A) (*p*-value=0.092) and Security Perceived (SP) (*p*-value=0.529). These results align with our initial hypothesis, indicating that the robot with personalised activity selection (Condition 1) is rated more positively than a robot with random activity selection (Condition 2). Contrary to what we expected, we did not find any significant statistical differences in Animacy (AN) (*p*-value=0.157).

**Table 5:** Results of the statistical analysis carried out on the categories of the Godspeed questionnaire. Significant statistical differences (*p*-value from 0.05 to 0.01) are denoted with *, very significant (*p*-value from 0.01 to 0.001) with **, and extremely significant (*p*-value$<0.001$) with ***

Goodspeed category	Students’ T	DoF	*p*-value
Anthropomorphism	1.767	5	.092
Animacy	1.471	6	.157
Likeability	6.197	5	.000***
Intelligence perceived	3.788	5	.000***
Security perceived	0.640	3	.529

## Discussion and limitations

One of the goals of social robots is to communicate with their users appropriately. These users may present very different features, including a diverse range of preferences. For this reason, if a robot aims at entertaining users during long-term interactions, it has to exhibit adaptive capabilities to suggest each user with their favourite activities. Currently, many machine learning algorithms allow fulfilling this goal. However, like presented in this manuscript, preference learning and, more precisely, LRF is nowadays a competitive alternative that allows artificial systems to predict, using rankings, the favourite activities of each user from a predefined set of alternatives. The review of the existing literature lacks research about this area, as most current models focus on learning while interacting instead of anticipating the potential preferences of users before the interaction occurs using their defining features. However, we took strong inspiration from the literature presented in Sect. 2, but including some novel methodology to overcome some issues that our social robot and architecture presented. Unlike related literature for preference predictions based on deep learning or reinforcement learning, our contribution uses Label Ranking combined with random forest for several reasons. First, our dataset is not large, as deep learning models require to produce accurate outcomes. Second, deep learning models require a higher computational complexity and learning times that our robot may not easily afford due to its hardware limitations. Finally, as mentioned above, already existing methods typically need that the user interacts with the system before the estimation occurs. For this reason, our framework pretends to overcome the previous limitations to improve human–robot interaction by personalising activity selection estimating the preferred user’s activities.

Due to the broad range of algorithms that provide meaningful results in LRF problems, we opted for comparing two promising but different alternatives: Top Label as Class and Ranking by Pairwise Comparison. We demonstrated how Ranking by Pairwise Comparison is the best alternative since it produces promising outcomes in our learning scenario, especially for datasets with a large number of items to rank. To prove the improvements of personalising the activity selection according to the user’s preferences, we present different case studies about the operation of the learning system in the long run. Besides, we conducted a short-term human–robot interaction experiment for assessing whether people prefer interacting with a robot presenting personalised activity selection (Condition 1) than with a robot with random activity selection (Condition 2). The statistical analysis carried out on the data retrieved from the study shows valuable outcomes in comparing both conditions. The participants believe that a robot with personalised activity selection is more appropriate to their preferences, as well as they perceive this robot as more intelligent, and they like it above the robot without personalised activity selection.

Although the previous results show optimistic and promising outcomes in the adaptive selection of activities by the robot and the correct operation of the system, the learning process of the robot has some limitations that are worthy of mentioning. In the first place, we are aware that retrieving the data using an online survey for building our datasets and training the model is not the best alternative. Nonetheless, due to the COVID-19 pandemic, we believed it was the safest way to obtain the necessary data. Besides, the collection of the input features for test users was carried out using the questionnaire presented in Appendix A. We are aware that the best idea is to give the robot the possibility to gather such information interacting with the user. For this reason, we are currently working on designing a new robot functionality that allows Mini to retrieve the input features of the users by interacting with them.

Another limitation of the system resides in comparing the two LRF algorithms used in this work. We selected them because previous preference learning scenarios suggested promising results, but many other alternatives could be selected. Besides, as Sect. 6.1.1 shows, hyperparameter optimisation is an arduous task since it requires much computational time. Although these issues, the results demonstrated that our initial decisions in terms of the technique used in our learning model were, at least, appropriate for the kind of task we wanted to carry out. Finally, as results in Table 2 prove, LRF algorithms are strongly affected by the number of labels to rank. In datasets with a high number of labels, such as the Music dataset in this work (7 items in the ranking), the ranking correlation value significantly drops for datasets containing three items to rank (e.g. Entertainment or Games). Besides, the computational time required for training the system exponentially increases too. Although this is not an issue in this application, we must be aware of these drawbacks if we continue expanding the entertainment repertoire of Mini.

The human–robot interaction experiment yielded encouraging results in evaluating the robot exhibiting a personalised activity selection. However, we are conscious that further experiments with more participants and during long-term interactions are required to see if the learning model works in the long run. The system presented in this work is just the first step in endowing social robots with personalised activity selection. In the future, we believe that the preference learning estimations should be combined with reinforcement learning to adapt the initial predictions to their real values using the feedback provided by the users. Finally, we pretend to enlarge the number of instances of the datasets by including the final ratings learnt by the reinforcement learning model to enhance the prediction accuracy and the estimated rankings of the original preference learning model presented in this dissertation.

## Conclusions

This contribution presents how the social robot Mini can predict the favourite activities of the potential robot users. Specifically, the adaptive decision-making system extends our previous research focused on using the robot during personalised interactions. First, we compared the performance of Ranking by Pairwise Comparison and Top Label as Class, two encouraging LR algorithms. The results demonstrate how Ranking by Pairwise Comparison outperforms Top Label as Class in our learning system, producing better overall estimations. Then, we present different case studies showing the operation of the architecture. Finally, a human–robot interaction study validates whether people prefer a personalised activity selection over a random one.

The preference learning system embedded in the decision-making of Mini includes different mechanisms of interaction depending on the user with whom it is interacting. The three case studies describe how the robot interacts with users with different proactivity levels, leading to broad and diverse communication between agents. Moreover, the decision-making system promotes activity exploration and user proactivity if necessary. In this context, we are aware that predictions, in certain situations, do not assure a perfect accuracy in preferences representation, especially whether users have not interacted previously with the robot or do not figure out how some of the activities work. Besides, sometimes users can change their interests in real time while interacting with the robot. To overcome these situations, this work embraces the first step towards preference learning modelling in social robots.

## Survey content

The following appendix contains the survey questions that were asked to users and their possible answers. For full details of the survey, please access the following link Online Survey. Sections A.1, A.2 and A.3 were used to obtain the input of the datasets, while user ratings in Sect. A.4 were used to shape the labelled output in the ranking format of each category and subcategory.

### Socio-demographical questions

1. Gender. Multi-choice question.
  - Male
  - Female
  - Other
2. Age. Open numerical question (range 1-99).
3. Nationality. Open question.
4. Education. Multi-choice question.
  - Primary School/Elementary education
  - Secondary School
  - Medium level vocational training
  - High level vocational training
  - Bachelors
  - Master or PhD.
  - Other
5. Occupation. Multi-choice question.
  - Qualified worker
  - Student
  - Unqualified worker
  - Unemployed
  - Other
6. Coexistence. Multi-choice question.
  - Alone
  - Couple
  - Familiar
  - Shared house
7. Place of residence. Multi-choice question.
  - Big city
  - Small city/Town
  - Village/Countryside
8. Physical activity. Multi-choice question.
  - Less than once a week
  - 1-3 times a week
  - 4-6 days a week
  - Everyday/Almost everyday

### Habits questions

1. Do you consider a proactive person? $1-5$ rating.
2. Do you daily make use of electronic devices to entertain yourself? Yes-No question.

### Interest questions

1. Indicate if you have interest in the following categories. Checkbox question.
  - Sports
  - Videogames
  - Reading
  - Photography
  - Information Search
  - Browse the Internet
  - Videos
  - Social Networks
  - Cooking
  - Purchases
  - TV series and films
  - Music

### Preferences ratings

Rate from $0 \; to \; 5$, meaning 0 total dislike and 5 total like the following entertainment categories, subcategories and activities.

1. Games
  - Classics
  - Calculus
  - Quiz
2. Photos
  - Monuments
  - Animals
  - Landscapes
  - Funny moments
3. Audiobooks
  - Classics
  - Historical events
  - Children’s tales
4. Music
  - Spanish rock
  - Classical music
  - English rock
  - Flamenco music
  - Spanish pop
  - Latin music
  - English pop
5. Videos
  - Sports
  - Comedy
  - Cooking
  - Film trailers
6. Sayings
7. Jokes
8. Information
  - Weather forecast
  - National news
  - International news
  - Sport news
  - Opinion articles
  - Breaking news
